# Knee complaints seen in general practice: active sport participants versus non-sport participants

**DOI:** 10.1186/1471-2474-9-36

**Published:** 2008-03-19

**Authors:** Marienke van Middelkoop, Robbart van Linschoten, Marjolein Y Berger, Bart W Koes, Sita MA Bierma-Zeinstra

**Affiliations:** 1 Department of General Practice, Erasmus MC, Rotterdam, The Netherlands

## Abstract

**Background:**

Since knee complaints are common among athletes and are frequently presented in general practice, it is of interest to investigate the type of knee complaints represented in general practice of athletes in comparison with those of non-athletes. Therefore, the aim of this study is to investigate the differences in type of knee complaints between sport participants, in this study defined as athletes, and non-sport participants, defined as non-athletes, presenting in general practice. Further, differences in the initial policy of the GP, medical consumption, and outcome at one-year follow-up were also investigated.

**Methods:**

Patients consulting their GP for a new episode of knee complaints were invited to participate in this prospective cohort study. From the total HONEUR knee cohort population (n = 1068) we extracted patients who were athletes (n = 421) or non-athletes (n = 388).

**Results:**

The results showed that acute distortions of the knee were significantly more diagnosed in athletes than in non-athletes (p = 0.04). Further, more athletes were advised by their GP to 'go easy on the knee' than the non-athletes (p < 0.01), but no differences were found in number of referrals and medication prescribed by the GP. The medical consumption was significantly higher among athletes; however, no significant differences were found between the two groups for recovery at one-year follow-up.

**Conclusion:**

There are no major differences in the diagnosis and prognosis of knee complaints between athletes and non-athletes presented to the GP. This implies that there are no indications for different treatment strategies applied in both groups. However, athletes are more often advised to 'go easy on the knee' and to rest than non-athletes. Further, there is a trend towards increased medical consumption among athletes while functional disability and pain are lower than among the non-athletes.

## Background

Complaints of the lower extremities are a serious problem because of their high prevalence and high impact on functional and work disability. A study among the Dutch general population showed a one-year prevalence of 21.9% for knee pain; about 33% of subjects reporting knee or hip complaints during the preceding year indicated that they had contacted their general practitioner (GP) for this complaint[1]. Among the Dutch population, knee problems are the most frequently presented complaints of the lower extremities: 21.4 per 1000 person-years for women and 22.8 per 1000 person-years for men[2].

Since sport activities are strongly promoted, the risk of sport injuries is likely to increase. Knee complaints are very common among sport participants[3,4] and it is reported that 39.8% of all sports injuries involve the knee[3]. Internal knee trauma, such as anterior cruciate ligament rupture, and distortion of the knee are the most common diagnoses of athletic knee injuries[3]. In addition, knee disorders such as the runner's knee, the patellofemoral pain syndrome, meniscus lesions and an anterior cruciate ligament rupture are often associated with sport participation[5,6].

In the Netherlands, almost everyone is registered in a general practice. At the time of conducting this study, all patients had first to visit their GP before being referred to a therapist or specialist in the Dutch health care system. Therefore, most care-seeking sport participants with knee complaints in the Netherlands will visit their GP for primary care. Since knee complaints are common among athletes and are frequently presented in general practice, it is of interest to investigate the type of knee complaints represented in general practice of athletes in comparison with those of non-athletes. These differences could have implications for applied treatment strategies of these knee complaints, i.e. it might be beneficial to treat the athletes different than the non-athletes because of a different diagnosis. Further, it is of interest to explore differences between athletes and non-athletes regarding the GP's initial treatment, medical consumption and prognosis of the two groups. If the medical consumption appears to be the only difference between athletes and non-athletes we will need to reflect on the implications of such difference.

Therefore, this study investigated differences in knee complaints between athletes and non-athletes presenting in general practice. The following questions were formulated: (1) Do athletes present with different knee complaints than non-athletes in general practice? (2) Is there a difference in initial policy of the GP between athletes and non-athletes? (3) Is there a difference in medical consumption between athletes and non-athletes during one-year follow-up? and (4) Do athletes have a better prognosis than non-athletes at one-year follow-up expressed in recovery, pain intensity and the WOMAC-score?

## Methods

### Study design

A prospective, observational cohort study was set up, with a follow-up of one year. A total of 40 GP's from 5 municipalities in the southwest region of the Netherlands (all connected to the Erasmus Medical Centre GP Research Network HONEUR) participated in this study. Recruitment of patients started in October 2001 and finished in October 2003. Patients aged 12 years and older, consulting their GP for a new episode of knee complaints were invited to participate in the study. Complaints that were presented to the GP for the first time, and recurrent complaints for which the GP was not consulted during the preceding 3 months, were considered to be new complaints. During such a consultation, the GP briefly informed the patients of the existence of the study and handed over written information and a baseline questionnaire. Interested patients forwarded their contact details to the researchers. The researchers contacted the patients to give additional information about the study, and to make an appointment to sign informed consent, and to perform a comprehensive standardized physical examination of both knees. GPs noted the working diagnoses of the knee disorders according to the International Classification of Primary Care. The consultations were taken in the same format as they usually take. Patient characteristics, medical history, knee history taking, GP's initial policy and sport activities were recorded in the baseline questionnaire. Follow-up questionnaires were sent to all participants at 3, 6, 9 and 12 months. Patients underwent a standardized physical examination at baseline and at one-year follow-up.

The researchers did not interfere with usual care with respect to advice, diagnostics or treatment. The Ethics Committee of the Erasmus Medical Centre Rotterdam approved the study. A detailed description of recruitment and data collection are reported elsewhere[7].

### Study population

**Figure 1:**
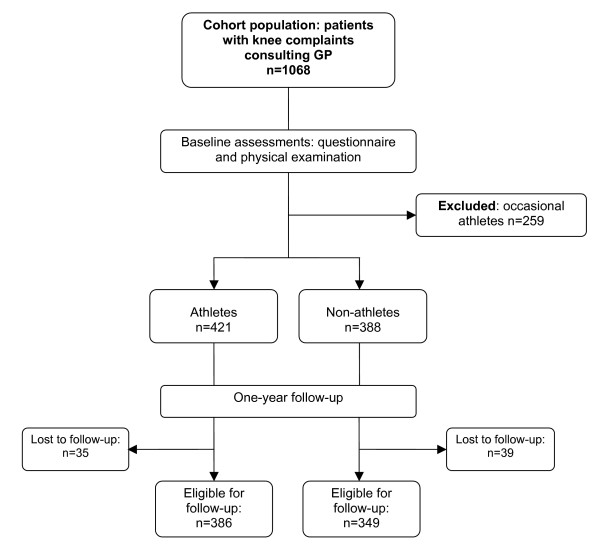
Flowchart.

A total of 1068 patients were recruited from 40 GP's (Fig. 1). From this total cohort population we extracted patients who were active sport participants, defined as athletes (n = 421) or non-sport participants, defined as non-athletes (n = 388). This selection was based on reported sport activities in the baseline questionnaire. Patients were first asked if they participated in any sport activity. Secondly, each patient could fill in his/her sport participation, to a maximum of three sports. For each sport activity, the type of sport, number of weeks of sport participation per year, and number of mean hours of sport participation per week were registered.

Athletes were defined as those who participated in sport for at least 30 weeks per year and minimally 2.5 hours a week for any one type of sport. Athletes who sport for minimally 20 weeks a year and at least 1.5 hours a week within one type of sport, and this for two or more sports, were also defined as athletes. The following activities reported on the questionnaires were not considered as being sport activities: bowls, billiards, darts, diving, golf, jeu de boules, go karting, 'slender you', shooting sports, fishing, and yoga. Non-athletes were defined as patients who reported no participation in sport activities at all. Because of the distinguishing power of this study, occasional athletes (n = 259) were excluded from this study (Fig. 1).

### Outcome measures

The four follow-up questionnaires reported on the medical consumption, pain, and functional disability of the knee of all participants. The medical consumption of the patients, expressed in frequency of visits, was calculated over the 12 months follow-up period. Pain was measured on a numerical rating scale (VAS) ranging from 0 (no pain) to 10 (unbearable pain). The WOMAC osteoarthritis index evaluates the functional disability of the knee with a score ranging from 0 (poor) to 100 points (excellent)[8,9]. After one-year follow-up, satisfaction with the GP's given policy, discomfort during employment and daily activities, and experienced recovery were registered. Patients' satisfaction was measured on an 11-point numerical rating scale from 0 (completely unsatisfied) to 10 (completely satisfied). Discomfort during employment and daily activities was measured dichotomously ("yes" or "no"). Experienced recovery was measured on a 7-point Likert scale ranging from total recovery (= 1) to worse than ever (= 7). The categories 'total recovery' and 'major improvement' represent a clinically relevant improvement and are in this study defined as being recovered. All other categories represent persistent knee complaints.

### Statistical analyses

Descriptive statistics were used to characterize demographic information, and chi-square and t-tests were applied to test the baseline differences for age, gender, BMI, WOMAC score and pain. Logistic regression analyses were used to test the association between athletic status and i) the type of knee complaint, ii) initial policy of the GP, iii) medical consumption, iv) patient satisfaction with GPs policy, v) recovery at one-year follow-up, and, vi) discomfort during employment and daily activities.

All of these analyse were adjusted for age, gender and BMI. In addition, models ii, iii, iv, v and vi were adjusted for trauma and baseline severity (measured by the WOMAC). Model vi was also adjusted for the appropriate baseline discomfort score.

Linear regression was used to test the association between athletic status and pain and function, as measured by the WOMAC. These analyses were adjusted for the potential confounders age, gender, BMI, trauma and baseline severity (WOMAC). The analyses for pain and function (WOMAC) were also adjusted for appropriate baseline pain and function scores, respectively.

The results of the logistic regression analyses are presented as odds ratios (ORs), with 95% confidence intervals (CI). A p-value less than 0.05 was considered significant. All analyses were performed with the SPSS software package (version 11.0, 2001).

## Results

### Study population

Comparison of baseline characteristics between dropouts (lost to follow-up at one year) and non-dropouts showed no significant differences with respect to gender, age) and the WOMAC score The pain score at baseline of the dropouts was significantly lower compared to the pain score of the non-dropouts (mean difference 0.69).

Table 1 presents baseline characteristics of the athletes and non-athletes.

**Table 1:** Baseline characteristics of the study population

		**Athletes (n = 421)**	**Non-athletes (n = 388)**	**OR (95%CI)**	**p-value**
Age (years)	Mean (SD)	41.0 (16.7)	50.0 (15.9)		**< 0.001**
Gender (male)	N (%)	244 (58.0%)	196 (50.5%)		**0.034**
BMI (kg/m^2^)	Mean (SD)	25.2 (4.1)	27.6 (4.9)		**< 0.001**
*Functional disability*					
WOMAC score	Mean (SD)	74.5 (19.5)	66.6 (21.1)		**< 0.001**
Pain (VAS)	Mean (SD)	4.20 (2.15)	4.46 (2.19)		**< 0.001**

Type of knee complaints*					

Trauma	N (%)	147 (34.9%)	111 (28.6%)	0.83 (0.60–1.14)	0.26
Bilateral	N (%)	19 (4.5%)	13 (3.4%)	0.96 (0.74–1.25)	0.76
Recurrent complaints	N (%)	159 (37.8%)	165 (42.5%)	0.84 (0.62–1.13)	0.24

Working diagnoses GP*					

General knee complaints	N (%)	138 (32.8%)	138 (35.6%)	0.93 (0.68–1.26)	0.62
Jumper's knee	N (%)	37 (8.8%)	38 (9.8%)	0.75 (0.46–1.24)	0.27
Acute distortion	N (%)	37 (8.8%)	20 (5.2%)	**1.92 (1.04–3.53)**	**0.04**
Osteoarthritis	N (%)	21 (5.0%)	49 (12.6%)	0.74 (0.42–1.33)	0.32
Osgood-Schlatter	N (%)	7 (1.7%)	1 (0.3%)	1.87 (0.22–16.1)	0.57
Acute meniscus/ligament rupture	N (%)	21 (5.0%)	17 (4.4%)	1.13 (0.57–2.25)	0.73
Chronic internal trauma	N (%)	45 (10.7%)	24 (6.2%)	1.66 (0.97–2.85)	0.07
Patellofemoral pain syndrome	N (%)	52 (12.4%)	37 (9.5%)	0.87 (0.54–1.40)	0.56
Chronic meniscus fracture	N (%)	5 (1.2%)	10 (2.6%)	0.54 (0.18–1.68)	0.29

* Analyses adjusted for gender, age and BMI. Significant differences are printed bold.

The mean age(SD) of the total study population (n = 809) was 45.3(16.9) years. The mean age of the athletes was significantly lower than the non-athletes. The total study population consisted of 440 men (54.4%) and the mean BMI was 26.3(4.7); the BMI of the athletes was significantly lower (25.2(4.1)) than the non-athletes (27.6(4.9)). The functional disability score at baseline (WOMAC score) showed a significantly higher outcome, indicating better functioning, among the athletes.

Among the athletes, cycling was the most commonly practiced sport (54.2%), followed by walking (24.2%), fitness (17.1%), soccer (15.4%) and tennis (13.1%). At baseline, 177 (21.9%) athletes practiced two types of sports, and 101 (12.5%) athletes practiced three types of sports.

### Type of knee complaints

The different type of knee complaints among the study population are listed in Table 1. About 32% of all knee complaints in both groups were traumatic injuries. Almost 30% of the athletes sustained this injury during a sport activity. In total, 50% of the athletes reported an association between their knee complaint and their sport activity.

The most frequently presented knee complaints in general practice are designated as general knee complaints: 32.8% among athletes versus 35.6% among non-athletes. The patellofemoral pain syndrome (11%) is also a relatively often-diagnosed knee complaint. The proportion of acute distortions showed a significant difference: 5.2% of the non-athletes was labelled as 'acute distortion' compared with 8.8% of the athletes (p = 0.04); however this difference is small. Osteoarthritis is also often diagnosed in general practice (8.7%). Osteoarthritis is more frequently seen among the non-athletes (12.6%) than among the athletes group (5.0%); however, there was no significant difference in frequency ratio in the adjusted analysis.

### GP's initial policy and medical consumption

The initial policy of the GP at baseline is shown in Table 2. Most patients were advised to 'go easy on the knee', to 'rest' and to 'wait and see'. More athletes were advised to 'go easy on the knee' (p = 0.002). More than 25% of the patients were referred to a therapist and almost 25% of all patients were prescribed medication. No statistical differences were found between the two groups regarding medication (p = 0.33), and referrals for additional diagnostic testing (x-rays) (p = 0.91) and to specialists/therapists (p = 0.61).

**Table 2:** Initial policy of the general practitioner at the first visit for knee complaints

**Treatment by GP**	**Athletes**	**Non-athletes**	**OR (95% CI)**	**p-value**
'*Passive strategy*'			1.64 (1.20 – 2.23)	**0.002**
Wait and see	24.5%	19.8%		
Rest	26.4%	21.1%		
Go easy on the knee	42.5%	30.9%		
Compresses	10.9%	9.3%		
*Active strategy*			1.20 (0.83 – 1.73)	0.33
Exercises	19.5%	13.9%		
Reduce body weight	3.1%	7.5%		
*Medication*			0.84 (0.59 – 1.20)	0.33
Medication	19.5%	28.1%		
Injection	-	1.0%		
*Referrals for diagnostics*	13.5%	19.8%	0.98 (0.64 – 1.48)	0.91
*Referrals to care givers*			1.09 (0.79 – 1.49)	0.61
Therapist	29.5%	23.5%		
Medical specialist	10.2%	12.4%		

Analyses adjusted for gender, age, BMI, trauma and baseline WOMAC-score.

Significant differences are printed bold.

Table 3 shows the medical consumption, expressed in numbers of patients visiting a specialist or paramedic. More than one third of the patients revisited the GP for their knee complaints; significantly (p = 0.03) more athletes revisited the GP than non-athletes, but the difference is small. A therapist or specialist was visited by 40.6% of the athletes versus 38.7% of the non-athletes (p = 0.045). However, when the analysis was adjusted for 'revisiting the GP', there was no longer a relationship between being an athlete and medical consumption (p = 0.20). Most patients visited a physiotherapist (30%) or an orthopedic surgeon (19%). The mean number of visits to the physiotherapist was 10.3(7.5) among the athletes versus 11.1(8.7) among the non-athletes.

**Table 3:** Medical consumption at one-year follow-up.

**Medical consumption**	**Athletes**	**Non-athletes**	**OR (95%CI)**	**p-value**
Revisiting visit general practitioner	36.8%	35.8%	1.43 (1.04–1.96)	**0.029**
Visit to therapist or specialist:	40.6%	38.7%	1.38 (1.01 – 1.88)	**0.045**
*Physiotherapist*	30.4%	29.6%		
*Specialist*	6.2%	4.4%		
*Rheumatologist*	0.0%	0.8%		
*Orthopaedic surgeon*	20.0%	18.0%		
*Revalidation specialist*	0.2%	0.0%		
Therapist Cesar/Mensendieck	0.7%	1.0%		

Analyses adjusted for gender, age, BMI, trauma and baseline WOMAC-score.

Significant differences are printed bold.

In general, patients were very satisfied with the GP's policy of their knee complaints. Almost 43% of the patients scored an eight or higher on the numerical rating scale. The mean score on the 11-point numerical rating scale, among the athletes was 7.2(2.6) versus 7.6(2.5) among the non-athletes (p = 0.90; OR 0.99, 95%CI 0.93–1.06). Patients who were referred to a therapist (physiotherapist, manual therapist or occupational therapist) were generally very satisfied with their treatment: 62.1% of the athletes scored an eight or higher on the 11-point numerical scale versus 66.0% of the non-athletes (p = 0.86; OR 1.01, 95%CI 0.90–1.13).

### Course and prognosis

**Figure 2:**
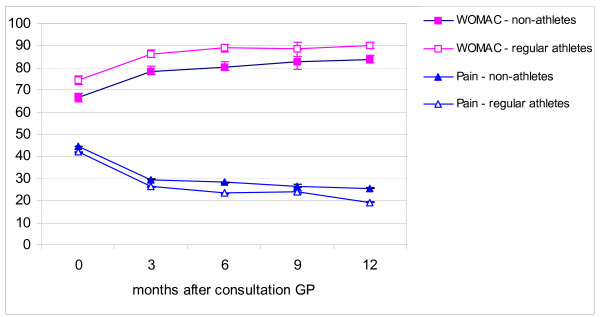
**Course of knee complaints (mean scores and 95% CI)**. Pain scores were multiplied with a factor 10 for graphical display.

Total recovery at one-year follow-up was reported by 59.8% of the athletes versus 50.7% of the non-athletes. However, self-reported recovery at one-year follow-up was not associated with being an athlete or not (p = 0.40; OR 1.15, 95%CI 0.83–1.58). Figure 2 shows the unadjusted mean pain and WOMAC scores at three-month intervals throughout one-year follow-up. The mean pain intensity scores of both groups decreased during follow-up. The mean pain score at one-year follow-up of the athletes was slightly lower than that of the non-athletes; however the difference was not significant (p = 0.20). The WOMAC functional disability score was higher during the entire follow-up among the athletes compared with the non-athletes; however, there was no significant difference at one-year follow-up between the two groups (p = 0.21).

About 10% of the athletes experienced discomfort during employment due to their knee complaints at one-year follow-up versus almost 15% of the non-athletes (p = 0.054; OR 0.62, 95%CI 0.38–1.01). The athletes also experienced less discomfort during any daily duties (17.6%)(employment, volunteer work, studies and housekeeping) compared to the non-athletes (29.6%) (p = 0.003; OR 0.56, 95%CI 0.38–0.83).

## Discussion

In this observational cohort study in general practice, most knee complaints were labelled as general knee complaints. Acute distortions were diagnosed significantly more often among athletes than non-athletes, but the difference was small. The GP advised more athletes to 'go easy on the knee' compared to non-athletes. Revisits to the GP occurred more frequently among athletes, and the athletes more frequently visited a therapist or specialist. At one-year follow-up the athletes experienced less discomfort during daily activities and employment due to their knee complaints than the non-athletes.

Because this is, to our knowledge, the first study comparing athletes and non-athletes with knee complaints, we cannot make any comparisons on this subject with current literature. However, in the present study, traumatic injuries were seen in almost 35% of the athletes and almost 30% of the traumatic injuries of this group were sustained during a sport activity. In total, 50% of the athletes associated their knee complaint with their sport participation. In subgroup analyses, there were no differences in the type of knee complaints between the athletes who associated their complaint with their sport participation and those who did not. This implies that there is no specific knee complaint that can be associated with sport participation.

Most studies on knees and athletes focus on knee injuries, which are mostly traumatic, whereas in our study only 35% of the knee complaints in athletes, presented in primary care, were traumatic. Therefore, future research should not only focus on traumatic knee injuries in athletes, but also on non-traumatic injuries.

Osteoarthritis was more often presented among the non-athletes: 12.6% among the non-athletes versus 5.0% among the athletes; however, the adjusted OR shows no significant difference. The difference between the two groups can probably be attributed to the differences in age, gender and BMI rather than to sport participation itself. The non-athletes were significantly older, had a higher BMI and included more females than the group of athletes. These latter findings are supported by other showing that higher age, BMI and female gender are associated with knee osteoarthritis [10-12].

Acute distortions were seen significantly more often in athletes (8.8%) than in non-athletes (5.2%), but the difference is small. Most of the distortions of the athletes occurred during soccer, cycling, fitness, tennis and walking.

There were few differences in the initial policy of the GP between the two groups. In this context it must be mentioned that there can be an overlap between the different treatment strategies, i.e. one patient could receive more than one advice and/or treatment at their first consultation at the GP. However, athletes were more often advised to 'go easy on the knee' than the non-athletes; this is probable related to the physical activity level of the athletes or to the type of knee complaints. Patients with acute distortions were significantly more often advised to 'go easy on the knee', whereas patients with osteoarthritis and chronic meniscus fractures were significantly less often advised to do this. These findings might also be related to the fact that it is difficult for non-athletes to reduce their level of physical activities.

In the Dutch healthcare system patients generally have to visit their GP before being referred to a therapist or specialist. Consequently, we found a strong relationship between revisiting the GP and medical consumption (p < 0.001). Therefore, we repeated the analysis for medical consumption (therapist or specialist) with adjustment for revisiting the GP. The adjusted analysis no longer showed a significant difference in medical consumption between the two groups (p = 0.20). Thus, referral to therapists or specialists in this study is more dependent on the number of GP visits than on being an athlete or not. In the adjusted analysis we also found a significant difference in revisiting the GP between the two groups. However, the difference between both groups is very small: 36.8% versus 35.8%. Analyses showed that revisiting GP is more dependent on age and functional disability at baseline than on being an athlete or not. It is however noteworthy that there is a trend towards increased medical consumption among the athletes while the functional disability scores are higher and the pain scores lower than among the non-athletes. Besides, the athletes experienced less discomfort during their daily and work duties than the non-athletes, which might indicate that the athletes make greater demands on their body than the non-athletes. The role of the GP in this relationship remains unknown, i.e. it is unknown if the GP is aware of the physical activity level of the individual patient at consultation.

At one-year follow-up, almost 55% of the athletes indicated that they had recovered from their knee complaint versus 45% of the non-athletes. This difference is, however, not significant (p = 0.40); the multivariate analysis showed that the recovery ratio is more dependent on age, gender and trauma than on physical activity level. Therefore, this study does not give any indications for the GP to inform athletes different than non-athletes regarding the prognosis of their knee complaints.

Finally, we did not find any substantial differences in the diagnosis and prognosis of the knee complaints between athletes and non-athletes but we did find a difference in medical consumption between the athletes and non-athletes. Apparently athletes do prefer a more active strategy compared to non-athletes. However, the exact reason for this higher medical consumption remains unknown.

### Limitations

More than one third of the knee complaints are labelled by the GP as 'general knee complaints', indicating some difficulty in arriving a precise diagnosis of the knee complaints of their patients.

Although the group of athletes consisted of more males and younger people, because all analyses were adjusted for age, gender and BMI this difference should have no impact on our final conclusions. Further, the physical workload of the patients might have influenced the results of this study. The baseline questionnaire included some questions about work tasks; unfortunately, this information was not sufficient to analyze this potential confounder.

## Conclusion

To our knowledge, this is the first study comparing athletes and non-athletes regarding knee pain in general practice. The results of this study indicate that there are no major differences in diagnosis and prognosis of knee complaints between athletes and non-athletes presented to the GP. This implies that there are no indications for different treatment strategies applied in both groups. Though, athletes are more often advised to 'go easy on the knee' and to rest than the non-athletes. However, this advice might be related to the physical activity level of the patients. Further, there is a trend towards increased medical consumption among athletes while the functional disability scores are higher and the pain scores are lower than among the non-athletes.

## Competing interests

The author(s) declare that they have no competing interests.

## Authors' contributions

SMABZ, BWK and MB conceived of the study, developed the design of this cohort study and contributed to the content of the article. MM carried out the statistical analyses and wrote the article. RL contributed to the content of this article and helped to draft the manuscript. All authors read an approved the final article.

## Pre-publication history

The pre-publication history for this paper can be accessed here:


